# Building meaningful relationships through co-design: reflections from a participatory multi-year research partnership to create MedManageSCI

**DOI:** 10.3389/frhs.2026.1876265

**Published:** 2026-09-07

**Authors:** Lauren Cadel, Angela Chow, Sander L. Hitzig, Lisa M. McCarthy, Shoshana Hahn-Goldberg, Carolen Carvalho, Emily Ching, Shannon Fiedorec, Michael Gateman, Anita Kaiser, Aaron Kowalchuk, Shveta Makim, Kiesha Mastrodimos, Holly Mo, Phillip Raney, Rasha El-Kotob, Sara J.T. Guilcher

**Affiliations:** 1 Leslie Dan Faculty of Pharmacy, University of Toronto, Toronto, ON, Canada; 2 Institute for Better Health, Trillium Health Partners, Mississauga, ON, Canada; 3 St. John's Rehab Research Program, Sunnybrook Research Institute, Sunnybrook Health Sciences Centre, Toronto, ON, Canada; 4 Rehabilitation Sciences Institute, Temerty Faculty of Medicine, University of Toronto, Toronto, ON, Canada; 5 Department of Occupational Science and Occupational Therapy, Temerty Faculty of Medicine, University of Toronto, Toronto, ON, Canada; 6 Dalla Lana School of Public Health, University of Toronto, Toronto, ON, Canada; 7 Department of Family and Community Medicine, Temerty Faculty of Medicine, University of Toronto, Toronto, ON, Canada; 8 Women's College Research Institute, Toronto, ON, Canada; 9 School of Pharmacy, University of Waterloo, Kitchener, ON, Canada; 10 OpenLab, University Health Network, Toronto, ON, Canada; 11 Toronto Rehab Lyndhurst Centre, University Health Network, Toronto, ON, Canada; 12 KITE Research Institute, University Health Network, Toronto, ON, Canada; 13 Vancouver Coastal Health, Vancouver, BC, Canada; 14 FunctionAbility Rehabilitation Services, Concord, ON, Canada; 15 Department of Physical Therapy, Temerty Faculty of Medicine, University of Toronto, Toronto, ON, Canada

**Keywords:** co-design, medication management, participatory research, patient engagement, spinal cord injury, toolkit design

## Abstract

**Background:**

Participatory approaches to research, including co-design, are essential to enhancing healthcare quality and safety and for minimizing the research-to-practice gap. Our team co-designed a medication self-management toolkit, MedManageSCI, to empower adults with spinal cord injury/dysfunction (SCI/D) to better integrate medications into their daily lives.

**Objective:**

We reflect on the lessons learned through the co-development of MedManageSCI and provide practical considerations for future co-design and implementation research.

**Methods:**

A participatory multi-methods study was conducted to co-develop, implement, and evaluate MedManageSCI. A working group comprised of adults with SCI/D, caregivers, and healthcare providers guided all aspects of this research, participating in a total of 10 meetings over the three-year project. In the final meeting, a reflective discussion was facilitated to identify key insights, practical strategies, and future considerations for engaging a diverse group of individuals in a multi-year, participatory research process. The audio-recording and notes from this session were analyzed independently and with the working group.

**Results:**

The working group consisted of nine adults with SCI/D, nine healthcare providers (e.g., physicians, nurses, pharmacists, physical therapists, occupational therapists), and one caregiver from across Canada. We identified an overarching theme, development of meaningful relationships, which was enabled through the following three key insights: co-creating a vision and having a collaborative culture, ensuring structured and intentional engagement, and closing the loop through authentic transparency. Across the overarching theme and insights, we discuss practical strategies and future considerations to support meaningful and sustained engagement in participatory research.

**Conclusions:**

By sharing our processes and key learnings, our goal is to inform and strengthen future co-design and implementation research by promoting shared and inclusive leadership, ensuring meaningful engagement, and fostering psychologically safe environments.

## Introduction

Translating research evidence into practice is essential for improving healthcare quality and safety (1); however, there are significant and persistent challenges in research being meaningfully integrated into real-world settings (2). This known knowledge-to-practice gap can be reduced by engaging people with lived experience (PWLE), healthcare providers, researchers, and other key interest groups throughout the research process (3). Engagement can occur across a continuum, including consultation (sharing information), involvement (providing input based on experiences or preferences), and partnership or shared leadership (actively shaping decisions or co-leading initiatives) (4). Despite many funding agencies increasingly requiring the inclusion of PWLE on research teams, PWLE are still frequently underrepresented as active partners compared to other key interest groups (i.e., healthcare providers), or have limited involvement in decision-making; this inequity is widespread across multiple health research domains, impacting knowledge, service change, and implementation (3). Given that PWLE are often the primary end-users of programs and interventions, this underrepresentation highlights an ethical concern and the need for co-design approaches to ensure that research outputs align with their priorities and needs (5, 6). Barriers to participation as research partners include limited funding, lack of time, limited organizational training and support for co-production and co-design, insufficient support for accessibility, systemic constraints, and prior negative experiences for individuals living with physical, neurological, and psychosocial disability, particularly concerning power and trust (7–10). PWLE who have participated on research teams have reported experiences of tokenism, feeling undervalued, emotional strain, excessive workloads, and negative attitudes from researchers and other key interest groups (11, 12).

In efforts to improve knowledge-to-action and encourage meaningful engagement, an effective co-design approach supports individuals throughout the process and respects their perspectives and values (13). Through co-design, key interest groups actively collaborate to develop solutions that are functional, meaningful, and beneficial for addressing complex healthcare challenges (13). Additional benefits of co-design include increased adoption and sustainability of developed resources, shared learning, and strong relationships between PWLE, researchers, and other key interest groups (13). While co-design approaches in research are increasingly being utilized, widespread gaps remain in knowledge and training on co-design processes, reporting, evaluation, and reflexivity (14, 15). The publication of reflexive accounts of co-design processes can support learnings about challenges, opportunities, and best practices for individuals, organizations, and lived experience co-design partners (16, 17).

Building on these co-design principles, over the past several years, we co-designed a toolkit to support adults with spinal cord injury/dysfunction (SCI/D) in managing their medications (18). Our team consisted of PWLE of SCI/D, researchers, healthcare providers, and caregivers from across Canada. SCI/D represents an example population with complex health and social needs (19). Adults with SCI/D commonly experience secondary conditions and take multiple medications (20–23). Unfortunately, there are limited resources available to support this population (24), so we set out to create a medication self-management toolkit in partnership with adults with SCI/D, caregivers, and healthcare providers from across Canada. The toolkit, MedManageSCI, was developed to address the core tasks (medical, emotional, and role management) and skills (problem solving, decision making, seeking formal and informal supports, goal setting, and engaging in activities) of medication self-management (18). In this paper, we reflect on the lessons learned through our partnership with key interest groups to co-design MedManageSCI. We also provide practical considerations for future co-design and implementation research.

## Materials and methods

### Research study context

This was a participatory multi-methods study conducted to co-design, implement, and evaluate a medication self-management toolkit for adults with SCI/D across Canada. The PRODUCES framework by Leask and colleagues was used to guide the participatory nature of the research, as it provides recommendations and principles specific to planning, conducting, evaluating, reporting, and adapting the co-creation of public health interventions (25).

The four stages of this study, previously reported elsewhere in greater detail, included: a concept mapping study to identify and prioritize content to include in the toolkit (26), a multi-methods study to create a prototype of the toolkit (18), a descriptive qualitative study to refine and finalize the toolkit (27), and a mixed methods pilot study to evaluate implementation outcomes and clinical/behavioural outcomes (28, 29). The study received ethics approval from the University of Toronto (#36588) and University of Alberta (#Pro00121103).

A working group comprised of adults with SCI/D, caregivers, and healthcare providers guided all aspects of this research. The working group was separate from the research team. Members of this working group were recruited through the distribution of physical and virtual flyers. We posted the information on our lab website and social media accounts and distributed it through our partner organizations’ newsletters. Working group members were emailed a document outlining the project goals, time commitment, and expectations. The working group met quarterly on Zoom to receive project updates and provide input, supported recruitment, provided guidance on data collection, data analysis, and materials for sharing findings, ensured the toolkit content was comprehensive, understandable and organized conceptually, and supported the dissemination of the toolkit and other study-related materials. Ten meetings were held between January 2023 and March 2026; eight of the meetings were with the full working group and two were with the adults with SCI/D, which focused on the design considerations. When members had differing views on key decisions, consensus was sought through discussion. If it could not be achieved, an anonymous poll was used to finalize the decision based on majority. A designated notetaker was present at each meeting to capture feedback and questions. Working group members received an honorarium in the form of a gift card of their choice after each meeting.

### Reflection methods

To identify key insights, practical strategies, and future considerations for engaging a diverse group of individuals in a multi-year, participatory research process, our research team reflected with the working group regarding our experiences in a final session. Prior to the session, examples of reflective questions were created by members of the research team (LC, AC, SJTG) and sent to the working group members by email (see Box 1). During the session, the reflective discussion was guided by a member of the research team (LC). The unstructured discussion allowed for members to build on the ideas of others and share alternative experiences or perspectives, while also allowing the facilitator to probe into different topics that were raised by the working group. This 45-minute session was audio-recorded and a notetaker (AC) was present to record the details of the discussion.

Box 1 Reflective Questions Guiding Final Working Group Session.

1. What are you most proud of in our work together?
2. What moments felt most energizing or meaningful?
3. What key strengths helped our group succeed?
4. What aspects of our process worked well? What could be improved?
5. Where did we create the most value or impact?
6. If we were to build on our strengths, what should we continue or expand?
7. Where could we do things differently to strengthen our work? Or improve your experiences as a member of the working group?

Data from the final working group session, inclusive of the audio-recording and detailed notes, were initially analyzed thematically by three members of the research team (LC, AC, SJTG). First, the team members independently analyzed the data to note key learnings and overarching ideas that contributed to the successful participatory process. A collaborative meeting was held amongst the three research team members to discuss the initial themes and key insights. A second meeting was held with a subset of individuals from the working group to further discuss the themes and key insights, including the processes and mechanisms that contributed to them, as well as to develop practical strategies and future considerations related to each. The themes, strategies, and considerations were updated based on feedback from the working group. Throughout the process, we ensured trustworthiness through member checking, peer de-briefing, and engaging in reflexivity (30).

## Results

### Working group members

The working group consisted of nine adults with SCI/D, nine healthcare providers (e.g., physicians, nurses, pharmacists, physical therapists, occupational therapists), and one caregiver. Six of the adults with SCI/D had traumatic injuries and three had non-traumatic injuries. Working group members were from three provinces in Canada: Ontario, Alberta, and British Columbia. The majority self-identified as women (*n* = 15), four identified as men, and none identified as transgender, non-binary, Two-Spirit, or gender expansive. Nine working group members self-identified as White and ten identified as other racial groups, such as South Asian, East or Southeast Asian, Indigenous, or mixed backgrounds. Most working group members had not been previously involved in a co-design project. The majority attended most meetings throughout the year and none formally withdrew over the three-year project.

### Key insights and practical considerations

**Figure 1:**
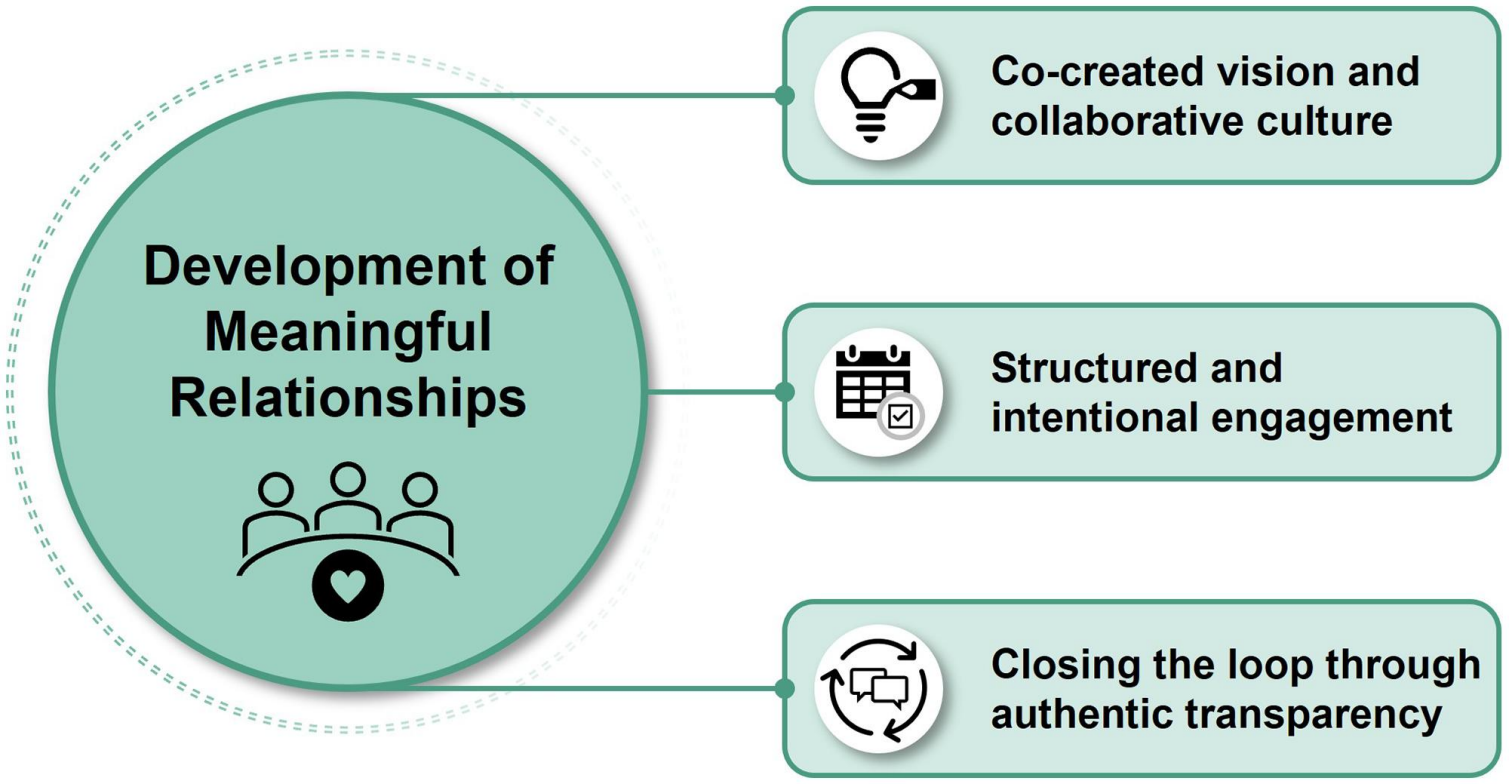
Overarching theme and key insights.

We identified an overarching theme, development of meaningful relationships, and three key insights: co-created vision and collaborative culture, structured and intentional engagement, and closing the loop (i.e., reporting back) through authentic transparency (see Figure 1). Working group members reflected on the mechanisms, structures, and processes that enabled meaningful interactions and relationships. The overarching theme and key insights, along with practical strategies and future considerations, are described below (see Table 1).

**Table 1:** Key insights, practical strategies implemented, and future considerations.

Key Insights	Practical Strategies (what we did)	Future Considerations (what we/ others can do)
Development of meaningful relationships	Engaged early and engaged often at a date and time that worked best for the majority of members (this supported retention and engagement over time) Offered to connect individually with working group members who were unable to attend group meetings Encouraged members to join virtual meetings from a comfortable location and with their cameras on to create more connection (if comfortable and appropriate) Built on pre-existing relationships that some PWLE of SCI/D had prior to our study	Host a pre-meeting prior to starting the project/study to allow members to get to know each other (e.g., icebreakers, activities, sharing fun facts) Circulate a list of working group members, including short bios and contact information to support connection and help refresh familiarity with names before meetings (if comfortable and agreed upon by group)
Co-created vision and collaborative culture	Established a diverse group of people from across Canada who brought individual experiences and perspectives, but who also had a collective commitment of enhancing medication self-management for PWLE of SCI/D Provided sufficient context and background to guide discussions, but did not over-structure and limit free-thinking (allowed co-creation to occur) Allowed members to build off the ideas and energy of others, which helped generate momentum and advance discussions and decisions	Ensure an experienced, open-minded facilitator leads the discussion and seeks input from all members Consider adding a co-facilitator who is a PWLE, key interest holder, or knowledge expert to support group discussions Allocate adequate time for group discussions, especially at key decision points Identify the adequate number and types of members based on project goals and length Have a pre-determined method for resolving disagreements and/or achieving consensus on key project decisions
Structured and intentional engagement	Developed and had working group members sign a formal letter that outlined the project scope, expectations, and time commitment Held regular, virtual meetings to ensure regular touchpoints for ongoing collaboration and project updates Offered multiple engagement and feedback options through group discussions, chat, individual meetings, and email Circulated a meeting agenda and materials in advance to allow members to review ahead of time and come to meetings prepared to provide feedback and engage in discussions Used a combination of open-ended questions and targeted follow-up questions to facilitate deeper discussion and clarify key points Compensated working group members for their time and contributions in a timely manner (gift cards to their preferred location)	If engaging virtually, confirm which platform is preferred by members Allow multiple methods for members to engage and provide feedback (e.g., group or individual discussions, written feedback, reactions on virtual platforms, polls) Ensure the facilitator is comfortable moderating discussions by providing training or practice sessions before the project
Closing the loop through authentic transparency	Engaged in active, non-judgmental listening to promote idea-sharing and collaborative discussion among the group Allowed working group members to support and build on the ideas of others Created an environment where all input was welcomed and valued (e.g., nothing was perceived as too small or trivial) Incorporated feedback and suggestions from working group and followed up to explain and demonstrate how input was addressed, or provided a clear rationale if recommendations could not be addressed	Provide clear and honest rationale when recommendations cannot be implemented (for both major and minor feedback) Take detailed notes about feedback and project decisions to keep working group informed

#### Development of meaningful relationships

Working group members reflected on their experience co-designing MedManageSCI, with some describing it as a good example of effectively engaging with PWLE. The multi-year timeline of this project supported relationship-building, allowing members to develop trust and form deeper connections over an extended time period. The team also discussed and reflected on the respect individuals had for one another. Working group members valued hearing different viewpoints and enjoyed learning about individual experiences, as it allowed for collective learning, but also enhanced the end result of the toolkit. Working together to create a practical resource resulted in a strong sense of pride in what was created. There were individuals within the group who naturally took on leadership roles as connectors, ensuring everyone felt welcome and had the space to share their views. It is important to note that some of the working group members with SCI/D had relationships prior to the research study, which may have increased their comfort with one another. In addition to the group interactions, the environment was described as genuine, engaging, and a safe place for contribution and learning, which is key for building and maintaining relationships. Ultimately, meaningful relationships within the working group and with those whose primary role was a researcher (LC, AC, RE, SJTG) were facilitated by co-creating a shared vision and having a collaborative culture, ensuring structured and intentional engagement, and closing the loop (i.e., reporting back) through authentic transparency.

#### Co-created vision and collaborative culture

Working group members valued the collaborative and iterative processes that shaped the development of MedManageSCI. They described how the toolkit evolved organically over time, with their ideas driving the overall direction. While the researchers had the goal of developing a resource to support adults with SCI/D with medication self-management, there was no pre-determined vision. Instead, working group members appreciated the opportunity to co-create the vision in partnership with the researchers, contributing to both the macro elements (e.g., what is being created, what content will be included, how will it be delivered) and the micro elements (e.g., fonts, colour scheme, toolkit name). The working group also valued contributing to the development of the mission, and values of MedManageSCI, which served as the foundational elements guiding its overall direction. The name, look, and feel of the toolkit were established through ongoing discussions, integrating diverse perspectives. The study followed a collaborative and iterative process, which was described as particularly meaningful, where the ideas, opinions, and experiences of individuals with SCI/D, caregivers, and healthcare providers were shared, listened to, and integrated into MedManageSCI. This supported retention over time as working group members reflected on feeling valued, that they were contributing to something transformative with the potential for long-term impact, and that they were not tokenized. The collaborative process ultimately enabled the co-creation of a toolkit that is functional, meaningful, relevant, and beneficial to the SCI/D community.

#### Structured and intentional engagement

Working group members reflected on several factors that they liked and that allowed for structured and intentional engagement, including the group size, meeting cadence, and platform used for virtual meetings. Early in the process, our team asked members how they wanted to work together and what they hoped to get out of their involvement. This information helped to inform how and when they were engaged. While the working group contained a total of 19 members, not all individuals were able to attend every meeting (average attendance was 12 members). Group polls were used to accommodate different schedules and time zones by selecting dates and times that worked for the majority of members. The size of the group was described as small enough to have meaningful discussions and large enough to ensure diversity of experiences and perspectives. In instances where most members were able to attend the meeting, breakout rooms were used for smaller group discussions of approximately five individuals. This enabled the exchange of dialogue and allowed for individuals to build on the ideas and perspectives of others, while still ensuring efficiency and productivity. After each meeting, a slide deck and summary were shared. Members who were unable to attend could provide feedback via email or through one-on-one sessions.

Meetings were held virtually to reduce barriers to participation and facilitate the involvement of individuals from across Canada. They were intentionally scheduled to coincide with the phases of the project. There was space (approximately three months) between the meetings to allow time for reflection, while still maintaining momentum and accomplishing project milestones. Project updates were provided by email in between the scheduled meetings, which was a recommendation from the working group. The group meetings were perceived as beneficial as they allowed for collaborative learning, idea exchange, and collective problem solving. We used Zoom for the meetings, which was preferred by the working group members with SCI/D; however, some healthcare providers noted their preference for MS Teams to align with what was used at their organization. In reflecting on meeting structure, the working group members recommended having a pre-meeting before starting a co-design or participatory study to serve as an icebreaker, allowing participants to share about themselves, their experiences, and their backgrounds. In doing so, this would create space for more informal interactions, which could help build comfort and familiarity among members; ultimately strengthening early engagement and fostering more open, collaborative participation throughout the process.

#### Closing the loop through authentic transparency

Closing the loop refers to the process of reporting back to the working group to explain how their input was used, including what changes were implemented and why some suggestions were not adopted. A key strength of this research was our collective commitment to genuine and authentic collaboration working together. A common reflection from the working group was feeling heard and valued throughout the process. This was ensured through an ongoing cycle of engaging with the working group, obtaining and reflecting on feedback, integrating recommendations, and closing the loop (i.e., reporting back) on any outstanding tasks. Working group members liked when they could see their input addressed in real time. To do so, our team would share our screen when seeking feedback on visual content, allowing individuals to immediately see their suggestions implemented and enabled members to build on the ideas of others. Contributions from the working group were highly valued and reflected in subsequent iterations of the toolkit, which built trust and sustained engagement over time. Feedback and critique were approached constructively, even at the micro level (e.g., changing the colour of a wheelchair in a video). If recommendations could not be integrated, there was a discussion with the rationale as to why it could not be addressed. Continued emphasis on closing the loop contributed to more authentic interactions and showed the working group how they were actively influencing the development of MedManageSCI.

## Discussion

We worked collaboratively with people from across Canada to co-design, implement, and evaluate a medication self-management toolkit, MedManageSCI. We highlighted how three insights supported the development of meaningful relationships, which was key for successful engagement of PWLE. Based on our experiences, we shared our learnings, key insights, practical strategies, and future considerations to help guide and support others who want to engage in meaningful co-design. We will discuss our findings within the context of two concepts: psychological safety and inclusive leadership.

In a group environment, psychological safety can be understood as “a shared belief that the team is safe for interpersonal risk taking” (p. 354) and is rooted in trust and mutual respect (31). Psychological safety allows individuals to feel comfortable sharing ideas, asking questions, and contributing to conversations without fear of judgement or negative consequences (32). Equity-denied populations, including those with disabilities such as SCI/D, often report feeling involved in research tokenistically, rather than through authentic and meaningful relationships (33). When partnering with key interest groups from diverse backgrounds to co-design solutions, having a psychologically safe environment becomes increasingly important as it promotes engagement and creativity (34). Within the context of co-designing MedManageSCI, evidence of a psychologically safe space was apparent as working group members displayed respect, shared dislikes and differing opinions, and remained involved over the three-year period. To help create this environment, we intentionally emphasized relationship-building early in the process. More specifically, we ensured consistent communication, promoted co-creation and development of a shared purpose, allowed time for informal discussions during sessions, and welcomed diverse perspectives and experiences. This focus on relationship-building may have contributed to the development of camaraderie and trust, as individuals learned that they would not be rejected for being themselves and that the team valued them as people (31). Ultimately, doing the groundwork to create meaningful relationships was critical not only for fostering psychological safety, but also for enabling authentic co-design.

Aligning with and essential for enabling psychological safety is the concept of inclusive leadership, which fosters a sense of uniqueness (e.g., supporting and empowering individuals, promoting diversity), creates a sense of belonging (e.g., ensuring equity, sharing decision-making, building relationships), shows appreciation (e.g., recognizing efforts), and promotes inclusion (e.g., creating an environment where individuals are heard) (35). Inclusive leadership has been shown to enhance psychological safety and positively influence innovative performance at the individual and team levels (36). While often used in work environments, inclusive leadership is also directly applicable to research environments as it is characterized by the leader's accessibility, availability, and openness (36). Building on these characteristics, it is imperative that leaders of co-design projects (e.g., researchers, principal investigators, knowledge users) show humility, invite idea sharing rather than direct solutions, and work *with* the co-design team to co-create a shared vision and purpose. We intentionally highlighted that members had varying perspectives and experiences and recognized that as a strength that would enhance MedManageSCI. Members of our working group also embodied inclusive leadership qualities by actively inviting diverse perspectives in discussions and fostering meaningful relationships within and outside designated working group sessions, which supported an engaging and collaborative environment. Ultimately, having a team of persons with lived experience with SCI/D, researchers, healthcare providers, and caregivers that was committed to working together was a key driver that enabled the successful co-design of MedManageSCI.

## Practical/ future considerations

Based on our reflections and learnings from co-designing MedManageSCI, we provided several recommendations for teams planning to engage in co-design or participatory research (see Table 1). Our practical strategies and recommendations align with the Integrated Knowledge Translation (IKT) Guiding Principles, which were created to support partnership projects by ensuring they are useful, useable, relevant, and not tokenistic (37). The principles emphasize building respectful and transparent relationships, fostering shared decision-making and open communication, valuing diverse expertise, remaining flexible in approach, ensuring meaningful benefits, and addressing ethical, practical, and financial considerations.

## Limitations

There are a few limitations to note. Our working group only contained one caregiver, meaning that the reflections shared in this paper may be more representative of those with SCI/D and healthcare providers. All members were from Ontario, Alberta, or British Columbia, with no involvement of individuals from other provinces and territories. Finally, working group members were required to be able to read and communicate in English, which may have limited inclusion of interested individuals who did not speak English.

## Conclusion

We reflected on key lessons and insights that enabled the successful co-design of MedManageSCI. This included co-creating a shared vision and collaborative culture, ensuring structured and intentional engagement, and closing the loop through authentic transparency, all of which contributed to the development of meaningful relationships. By reflecting on our processes and key learnings, the goal is that these insights can inform and strengthen future co-design and implementation research by promoting shared and inclusive leadership, ensuring meaningful engagement, and fostering psychologically safe environments.

## Data Availability

The raw data supporting the conclusions of this article will be made available by the authors, without undue reservation.
